# Nanoliposomal Encapsulation and Purification of Angiotensin-Converting Enzyme Inhibitor Peptides from Ulva rigida

**DOI:** 10.1021/acsomega.5c00780

**Published:** 2025-05-16

**Authors:** Eda Şensu, Harun Koku, Evren Demircan, Sebahat Şişman, İbrahim Gülseren, Tuğçe Karaduman, Bilal Çakır, Emine Şükran Okudan, Gökhan Duruksu, Beraat Özçelik, Aysun Yücetepe

**Affiliations:** † Department of Food Engineering, Faculty of Chemical and Metallurgical Engineering, 52971 Istanbul Technical University, Maslak, TR-34469 Istanbul, Türkiye; ‡ Department of Food Technology, Istanbul Gelisim Vocational School, Istanbul Gelisim University, 34310 Istanbul, Türkiye; § Department of Chemical Engineering, Faculty of Engineering, 52984 Middle East Technical University, Dumlupınar Bulv. No. 1, 06800 Ankara, Türkiye; ∥ Department of Food Engineering, 226843 Istanbul Sabahattin Zaim University, Halkalı-Kuçukcekmece, 34303 Istanbul, Türkiye; ⊥ Department of Molecular Biology and Genetics, Faculty of Science and Letters, 175169 Aksaray University, TR-68100 Aksaray, Türkiye; # Faculty of Fisheries, 37502 Akdeniz University, Dumlupınar Bulvarı, 07058 Antalya, Türkiye; ∇ Stem Cell and Gene Therapy Research and Applied Center, 52980 Kocaeli University, 41380 Kocaeli, Türkiye; ○ Department of Food Engineering, Faculty of Engineering, Aksaray University, TR-68100 Aksaray, Türkiye

## Abstract

Angiotensin-converting enzyme inhibitory peptides derived from natural sources may be effective in the treatment of hypertension without causing side effects compared with existing angiotensin-converting enzyme (ACE) inhibitors. Naturally derived antihypertensive peptides are therefore considered a promising alternative for the prevention or treatment of hypertension. Therefore, the study aimed to purify and identify ACE-inhibitory peptides from the green macroalgae Ulva rigida. In addition, the encapsulation of the purified peptides showed the highest ACE-inhibitory activity by chitosan-coated nanoliposomes, and the characterization of nanoliposomes was evaluated. Protein hydrolysates were obtained from U. rigida through enzymatic hydrolysis. The hydrolysates were separated into molecular weights of <3, <5, and <10 kDa through ultrafiltration membrane separation (UFMS). The <3 kDa fraction (UFMS-3) that exhibited the highest ACE-inhibitory activity (77.02%, 1 mg/mL) was purified using ion-exchange chromatography. Fraction-1 (IEC-F1) obtained from the ion-exchange purification showed an impressive 82.03% ACE-inhibitory activity. Moreover, peptide sequences of IEC-F1 were identified by LC-MS/MS, and their bioactive properties were determined *in silico*. After that, IEC-F1, with a strong ACE-inhibitory activity, was loaded into chitosan-coated nanoliposomes to improve their stability for encapsulation. Physical stability (ζ-potential, polydispersity index, particle size), thermal (DSC) and morphological properties (SEM), and FT-IR analyses were carried out for the characterization of nanoliposomes. Encapsulation efficiency was found to be 92.0 ± 4.5%. After encapsulation, the ACE-inhibitory activity of IEC-F1 was protected by 37.5%. Overall, the obtained findings indicate that the hydrolysate produced by the successive hydrolysis of U. rigida macroalgae with pepsin and trypsin contains peptides with strong ACE-inhibitory action. Furthermore, the chitosan-coated nanoliposome method was determined to be an effective carrier for the delivery of peptide fractions, showing ACE-inhibitory activity. The formulation of chitosan-coated nanoliposomes for peptide fractions from U. rigida represents an innovative approach that allows the development of functional and stable products.

## Graphical abstract

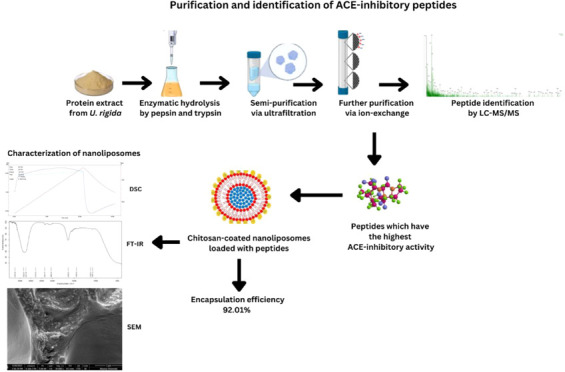

## 1 Introduction

Hypertension, or high blood pressure, is the leading cause of cardiovascular disease worldwide.^1^ The World Health Organization has identified hypertension as the predominant risk factor for mortality and morbidity since 2003.^2^ The renin-angiotensin system (RAS) is a physiological regulatory mechanism that controls blood pressure. The RAS involves the conversion of angiotensin (AT)-I to AT-II. The mechanism of this conversion is explained by the cleavage of AT-I at the histidyl residue from the C-terminus by the activity of ACE. This results in the production of AT-II. AT-II, a potent vasoconstrictor, plays a critical role in maintaining normal blood pressure. Inhibitors of ACE, an essential component of the RAS pathway, have been employed as antihypertensive agents. The mechanism of ACE inhibition involves competitive inhibition, wherein the peptides compete with the substrate for the enzyme’s catalytic sites. In addition, some peptides have been observed to exhibit uncompetitive inhibition, in which the peptides bind to other enzyme sites, resulting in alterations to the enzyme’s conformation and a subsequent decrease in its activity.^3^ For this purpose, synthetic ACE-inhibitors such as benazepril, captopril, enalapril, alacepril, and many others are frequently used to treat hypertension.^4^ Despite their efficacy, synthetic ACE-inhibitors are associated with documented side effects such as cough and skin rashes.^5^ Therefore, peptides from natural sources can be used as alternatives for pharmaceuticals and synthetic drug candidates.

Bioactive peptides (BAPs) are amino acid sequences ranging from 2 to 20 residues and encoded as specific peptide sequences in the primary structure of plant and animal proteins.^6^ Most bioactive peptides are produced by *in vitro* enzymatic hydrolysis or fermentation. BAPs are initially inactive within the primary structure of plant and animal proteins but can become active through processes such as enzymatic hydrolysis. These peptides, often released via protein hydrolysis, exhibit greater bioactivity than their parent proteins, underscoring the significance of peptide bond breakdown in unlocking their potential.^7^

Enzymatic hydrolysis is performed using one or more proteases to release bioactive peptides from food proteins that are desired to be released.^3^ BAPs show hormone- or drug-like activities and can be classified according to their mode of action as antimicrobial, antithrombotic, antihypertensive, opioid, immunomodulatory, mineral binding, and antioxidative.^8^ Regardless of the health benefits of bioactive peptides, ambient conditions such as low pH values can lead to the denaturation of bioactive peptides during gastrointestinal digestion and food processing.^9^ In addition, the bitter taste of bioactive peptides limits their use in food applications. Therefore, encapsulating bioactive peptides is necessary to enhance their stability, conceal their unpleasant taste, and increase shelf life.^10,11^

Encapsulation can be defined as the confinement of active substances within the wall materials. In the food industry, encapsulation protects bioactive ingredients and their effective distribution in foods.^12^ The primary methods for encapsulating bioactive peptides include liposomes, spray drying, double emulsion, freeze-drying, emulsification–gelation, and ionic gelation.^13^ Nanoliposomes are commonly used for loading, protecting, and releasing bioactive compounds because they are made from edible materials. Liposomes are spherical nano- or microscale vesicles made of phospholipid bilayers that provide controlled release, low toxicity, biocompatibility, and targeted delivery, making them valuable for encapsulation.^14^ They can carry hydrophilic compounds within their aqueous core and lipophilic compounds within the lipid bilayer, enabling the encapsulation of diverse bioactive substances, including ACE-inhibitory peptides with high hydrophobic amino acid content.^15,16^ This encapsulation stabilizes BAPs in acidic environments, such as the stomach, enhancing their bioavailability in the gastrointestinal tract. Chitosan coating optimizes nanoliposomal performance by providing a protective layer that increases nanoliposome stability, enables controlled release, and improves biocompatibility.^17^ The study by Forutan et al.^18^ showed that chitosan-coated nanoliposomes loaded with hydrolyzed protein from Spirulina platensis increased size and ζ-potential, improved stability, and reduced protein release.

Macroalgae are highly interesting natural sources of bioactive peptides due to their protein content. It has been accepted that bioactive peptides isolated from macroalgae have positive health effects due to their low molecular weight.^19^ Smaller peptides are more easily absorbed from the digestive tract into the bloodstream, allowing them to cross biological membranes more easily than larger proteins. This improved bioavailability means that the digested peptide reaches its target tissues and exerts its intended effect. Low-molecular-weight peptides can fit more easily into the binding sites of cell surface receptors, allowing them to interact effectively with their target receptors, thus contributing to their biological activity.^20^

*Ulva* spp., a member of the *Chlorophyceae* family, is one of the most common edible green algae.^21^ *Ulva* spp. is a valuable natural resource with commercial potential in human and animal nutrition. Ulva rigida has a large biomass and excellent nutritional composition (proteins 17.8%, fat 0.9%, carbohydrate 54.5%, and ash 28.6%).^22^ Various bioactive and nutritious compounds have been reported in U. rigida. It includes natural pigments, phenolic compounds, polyunsaturated fatty acids, lipids, proteins, and polysaccharides. These bioactive compounds are believed to have many potential health benefits that can be exploited in the nutraceutical and cosmetic industries.^23^ In this context, while ACE-inhibitory peptides derived from U. rigida proteins have been previously characterized,^24^ there are currently no reports on developing nanoliposomal-encapsulated peptide inhibitors from this source.

To the best of our knowledge, this is the first study on the purification, identification, and encapsulation of ACE-inhibitory peptides from U. rigida macroalgae collected from the Aegan coast of Türkiye. The aims of the study were as follows: (1) to separate and purify bioactive peptides from U. rigida by applying ultrafiltration membrane separation and ion-exchange chromatography, subsequently (2) observe the ACE-inhibitory activity of bioactive peptide fractions, (3) to identify peptide sequence(s) of the fraction by LC-MS/MS, (4) to analyze *in vitro* cytotoxicity of ACE-inhibitory peptide fractions, (5) to encapsulate the ACE-inhibitory peptides by using chitosan-coated nanoliposomal encapsulation method, and (6) to investigate the impact of chitosan coating on physical (ζ-potential, particle size) and thermal properties (DSC), encapsulation efficiency, chemical structure (FT-IR), and morphology (SEM) of the nanoliposomes that contain the peptide fractions.

## 2 Materials and Methods

### 2.1 Materials

U. rigida (Ulvaceae, Ulvales, Chlorophyta), a green macroalgae, is a widely distributed green seaweed that thrives in a variety of salinity and temperature conditions, frequently creating huge blooms (“green tides”) in nutrient-rich environments. Its great photosynthetic efficiency and flexibility make it a valuable bioindicator for detecting eutrophication in aquatic ecosystems. U. rigida was collected from the Aegean coast (40°14′27.03″N 26°32′29.74″E) of Türkiye by free diving at depths between 0 and 3 m (Figure 1). The collected macroalgae were preserved in 4–6% neutralized formaldehyde solution for identification and classified according to their morphological characteristics using stereo zoom (Olympus, SZX16) and binocular light (BX51) microscopes. The identified macroalgae were dried in a shaded area after removal of impurities, ground, and sieved to obtain powders <500 μm in diameter and then stored at −20 °C.

**Figure 1:**
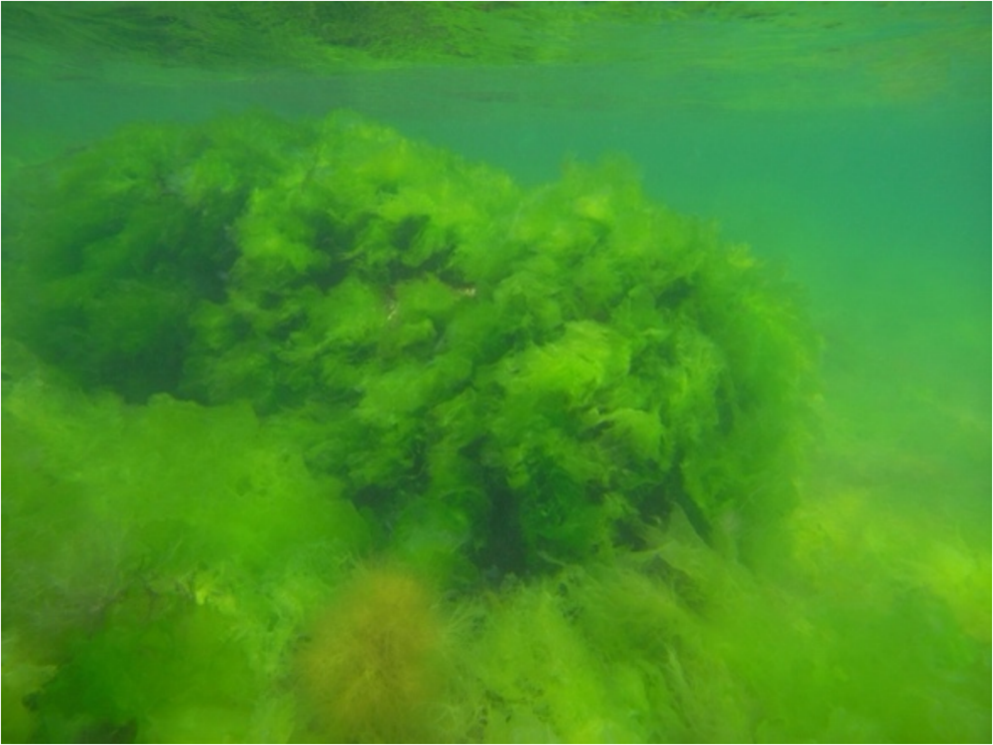
Submerged image of U. rigida by Dr. Emine Şükran Okudan.

Mouse fibroblast line L929 (Hukuk, Alum Institute, Ankara, Türkiye) was used for *in vitro* cytotoxicity analysis. Triton × 100 was obtained from Carl Roth GmbH (Karlsruhe, Germany). Lecithin (phospholipids from soybean with 70% phosphatidylcholine, Lipoid, Germany) and chitosan (Type B, medium molecular weight) were used for nanoliposomes. All solvents and chemicals were provided by Sigma-Aldrich (Sigma-Aldrich Co., St. Louis, MO) and were of the analytical grade.

### 2.2 Preparation of Enzymatic Hydrolysates

U. rigida protein extracts (URPE) obtained in our previous studies^23^ were subjected to sequential enzymatic hydrolysis with pepsin and trypsin. The hydrolysis method closely followed the protocol outlined by Ahn et al.^25^ and Cian et al.^26^ with minor modifications. First, U. rigida protein dispersion in distilled water (1%, w/v) was adjusted to pH 2 for the pepsin enzyme at an enzyme/substrate (E/S) ratio of 0.3:1 (w/w). Pepsin hydrolysis was performed in a shaking water bath (N-Biotek-303, Biotek Co., Ltd. Korea) at 75 rpm, 37 °C for 2 h. After pepsin hydrolysis, the pH of the medium was adjusted to 8, trypsin was added at an E/S ratio of 0.3:1, and incubation was performed under the same conditions. Afterward, the mixture was brought to 85 °C for 5 min to ensure enzyme inactivation. Following this step, the mixture was subjected to centrifugation at 3000*g* and 4 °C for 20 min. The protein content of the resulting supernatants was quantified using a Lowry method-based technique, as outlined by Lowry et al.^27^ The samples were stored at −20 °C for subsequent analyses. The ACE-inhibitory activity of the hydrolysate was performed.

### 2.3 Determination of Degree of Hydrolysis

The degree of hydrolysis (DH) was calculated according to the procedure of Vastag et al.^28^ In brief, the hydrolysates and TCA (0.44 M) were combined in a 1:1 (v/v) ratio and kept at 4 °C for 30 min. The solution was then centrifuged at 3000*g* for 20 min at 4 °C. The Lowry method was used to determine the protein content of both the 0.22 M TCA-soluble protein fraction and the fraction without TCA. Equation 1 was used to calculate the degree of hydrolysis.

1 $$\mathrm{DH}\%=\left(\frac{\mathrm{TCA soluble proteins}}{t\mathrm{otal proteins}}\right)\times 100$$

### 2.4 Separation and Purification Steps

#### 2.4.1 Ultrafiltration Membrane Separation

U. rigida protein hydrolysates (PH) were fractionated via ultrafiltration membrane separation (UFMS) using a lab-scale UFMS system (Sartorius Vivaflow 200, Germany) supplied with 3, 5, and 10 kDa cutoff membranes supplied by the same company (part numbers: VF20P9, VF20P1, and VF20P0, respectively), and they were coded as UFMS. After UFMS, three fractions were obtained (<10 kDa molecular weight (MW), <5 kDa MW and <3 kDa MW). The fraction with the strongest ACE-inhibitory was further purified using ion-exchange chromatography.

#### 2.4.2 Ion-Exchange Chromatography

The UFMS fractions (<3 kDa MW) were subjected to further purification using ion-exchange chromatography (IEC). AKTA Prime system (Amersham Biosciences, Sweden) outfitted with a HiPrep Q XL 16/10 column, a powerful anion-exchanger prepacked with Q Sepharose XL columns for initial protein and biomolecule capture using ion-exchange chromatography and PrimeView 1.0 monitoring software was used for this purpose. The mobile phase was 0.05 M phosphate buffer at pH 7.5, and the flow rate was adjusted at 1 mL/min. For elution, 1.0 M NaCl was introduced as a step input series of 0, 25, 50, 75, and 100% by volume (as a percent of buffer volumetric flow rate) to the column, and the resultant fractions were collected. The eluting peaks were identified by UV absorbance at 220–280 nm. The fractions with the highest ACE-inhibitory activity were identified among the collected fractions.

### 2.5 Characterization of Peptides

#### 2.5.1 Peptide Identification

Peptide identification was carried out using Waters Acquity UPLC I-Class Plus LC System and Xevo TQ-XS Mass Spectrometer supplied with a BEH C18 column (2.1 mm × 50 mm × 1.7 μm). The peptides were separated by passing 0.5 mL/min with an injection volume of 5 μL. The column and sample temperatures were kept at 40 and 10 °C, respectively. Mobile phase A was prepared using an aqueous solution containing 0.1% (v/v) formic acid, while mobile phase B was formulated with 0.1% (v/v) formic acid in acetonitrile. The solvent system as a gradient in the 15 min program is as follows: 0–5 min: 15% B; 5–8 min: 25% B; 8–10 min: 50% B; 10–12 min: 85% B; 12–15 min: 15% B. The MS settings were adjusted as 60 V sample cone voltage, 0.5 kV capillary voltage, and 20–40 eV collision energy.

##### 2.5.1.1 *In Silico* Analysis

The bioactive properties of peptide sequences were validated by the *in silico* method. PeptideRanker score was used to evaluate the bioactive potential of peptides. The PeptideRanker algorithm aims to identify new bioactive peptides by evaluating the general properties of peptides based on their structural similarity. The potential ACE-inhibitory activity of the peptides was determined with the BIOPEP “Calculations” tool (http://www.uwm.edu.pl/biochemia/index.php/pl/biopep).^29,30^

#### 2.5.2 *In Vitro* Cytotoxicity Analysis

For the evaluation of *in vitro* cytotoxic activity of the hydrolysate, obtained by the ion-exchange fraction with the highest ACE-inhibitory activity, a healthy mouse fibroblast line L929 was cultured in high-glucose DMEM medium containing 10% FBS and 1% penicillin/streptomycin. Hydrolysate fractions were sterilized with a 0.2 μm polycarbonate filter and prepared with DMEM medium to final concentrations of 125, 62.5, and 31.25 μg/mL.

Determination of cell viability was performed by using the MTT assay, which measures the activity of mitochondrial dehydrogenase in cells. First, 1 × 10^4^ cells were seeded in 96-well plates, and the plates were incubated at 37 °C in a 5% CO_2_ atmosphere. After 24 h of incubation, 10 μL of MTT solution (0.5 g/mL final concentration) was applied to each well. The medium of the control wells with serial dilutions prepared with solvent aliquots from the peptide dispersions was also replaced with fresh medium. After 24 h of incubation, 0.5 mg/mL MTT solution (2.5 and 5 g/mL peptide concentrations) was applied to each well. Following 3 h of incubation, the formation of formazan crystals was checked, and when present, the crystals were dissolved by adding 100 μL DMSO to the wells, and the resulting color intensity was measured spectrophotometrically with a ChroMateELISA reader at 492 nm. Wells containing only medium/solvent without peptide dispersions served as controls, and the percentage of cell viability was determined by the following equation in comparison to the control group.

2 $$c\mathrm{ell viability},\%=\left[\left(A_{\mathrm{sample}}\right)/\left(A_{\mathrm{control}}\right)\right]\times 100$$

where *A*_sample_ and *A*_control_ express the absorbance with and without peptide dispersions, respectively.

### 2.6 Angiotensin-Converting Enzyme–Inhibitory Activity

The *in vitro* ACE-inhibitory activities of the peptide dispersions were assayed by adapting the procedure used by Martinez-Alverez et al.^31^ Briefly, a 5 mM HHL substrate or ACE (100 mU) was prepared in 100 mM sodium phosphate buffer (pH 8.3) containing 300 mM NaCl. Then, 200 μL of the HHL solution and 50 μL of the samples were mixed and incubated at 37 °C for 10 min. Following the initial incubation, 20 μL of ACE enzyme dispersion was added to the mixture and incubated for 60 min at 37 °C in a shaking water bath. The enzymatic reaction was stopped by adding 250 μL of 1 M HCL. Subsequently, the quantification of released HA was carried out using HPLC. The ACE-inhibitory activity was quantified by an HPLC system (SPD M20A, Shimadzu) supplied with an analytical C18 column (4.6 × 150 mm × 5 μm). The sample was separated by passing 0.8 mL of eluent per min, and an injection volume of 10 μL was used. Aqueous 0.1% (v/v) TFA (eluent A) and 0.1% (v/v) TFA prepared in acetonitrile (eluent B) were used as the mobile phases. A linear gradient flow of 0–20% B was applied to the column for 5 min, followed by 20–60% B for the next 15 min. Immediately before completion, isocratic elution was maintained at 60% B for 4 min, and then, the system was restored to the initial eluent composition of 20% B. Elution peaks of HA and HHL were detected at 228 nm. The ACE-inhibitory activity (%) was calculated as follows:

3 $$\mathrm{ACE inhibitory activity}\;\left(\%\right)=\left(1-\frac{A_{\mathrm{sample}}}{A_{\mathrm{control}}}\right)\times 100$$

where *A*_sample_ and *A*_control_ express the relative areas (*A*) of the HA peak of the assays performed with and without ACE-inhibitors, respectively.

### 2.7 Encapsulation of Peptides

#### 2.7.1 Preparation of Nanoliposomes

The thin-film hydration method was performed to prepare nanoliposomes according to the procedure of Ma et al.^32^ In brief, lecithin (0.45 g), cholesterol (0.05 g), and Tween-80 (0.1 g) were dissolved in absolute ethanol (15 mL) for 15 min. After that, 2.5 mL of the peptide dispersion obtained with ion-exchange chromatography (0.14 mg/mL peptide concentration) was slowly added to the lecithin mixture, and the mixture was mixed using a magnetic stirrer (100 rpm). The mixture was subjected to homogenization using a shear mixer (Ultra-Turrax IKA T18, Janke & Kunkel, Staufen, Germany) at 5000 rpm for 1 min. Subsequently, ethanol in the mixture was evaporated using a rotary evaporator at 150 rpm and 40 °C until a thin layer of film was formed in the round-bottom flask. The thin lipid film was hydrated using 25 mL of phosphate buffer (0.005 M, pH 6.8). The suspension was stirred for 10 min on the magnetic stirrer, and it was homogenized by using the shear mixer under the same conditions. The reduction of particle size was performed by 10 cycles of sonication (Sonopuls HD 2200, Bandelin Electronic GmbH & Co., KG, Berlin, Germany), 1 min on at 20 kHz, then 1 min off under cold conditions (∼6–8 °C).

To prepare the chitosan coating dispersion, a chitosan solution (0.6% (w/v)) in 1% acetic acid (v/v, pH 3.5) was stirred for 16 h at room temperature. Then, the chitosan solution was slowly added to the nanoliposomal dispersion at a ratio of 1:1 (v/v) while stirring. The coated nanoliposomes were stored at 4 °C until further analysis, and the analysis was completed in 2 days. ACE-inhibitory activity, FT-IR, and physical stability assays were performed using the chitosan-coated nanoliposome dispersion.

Furthermore, for SEM and DSC analyses, a chitosan-coated nanoliposome dispersion was lyophilized (Christ α 1–2D plus, Germany). During the lyophilization process, sucrose as a cryoprotective agent was used to prevent the negative effects of lyophilization on nanoliposomes.^33^

### 2.8 Characterization of Nanoliposomes

#### 2.8.1 Encapsulation Efficiency

To determine encapsulation efficiency (EE), 5.0 mL of nanoliposomes was ultrafiltered using a 3 kDa molecular weight cutoff tube and centrifuged (2255*g*, 40 min).^10^ The concentration of free peptides (unloaded) in the permeate was measured using the Lowry method.^27^ Finally, the percentage of EE was calculated by using the following equation.

4 $$\mathrm{EE}\;\left(\%\right)=\frac{a\mathrm{mount of encapsulated peptides}}{t\mathrm{otal peptide concentration}}\times 100$$

#### 2.8.2 ζ-Potential, Particle Size Distribution, and Polydispersity Index

Zeta (ζ) potential was assessed using a particle charge titration analyzer (Stabino, Microtrac Europe, Montgomeryville, PA), while the particle size distribution and polydispersity index (PDI) were determined with a static light scattering instrument (Mastersizer MS2000, Malvern Instruments, Worcestershire, U.K.).

#### 2.8.3 Differential Scanning Calorimetry

Thermal properties of nanoliposomes were determined by differential scanning calorimetry (DSC, 60 Plus, Shimadzu Instruments, Japan). Briefly, 20 mg of nanoliposomes was placed in aluminum capsules, where empty aluminum capsules were used as a reference. The heating procedure was carried out between 25 and125 °C at a heating rate of 10 °C/min.

#### 2.8.4 Fourier Transform Infrared Spectroscopy

Organic groups in the nanoliposomes were determined using the Fourier transform infrared spectroscopy (FT-IR) technique with a Bruker Tensor II FT-IR spectrometer equipped with the ATR diamond module (Bruker Optics, Germany). All of the spectra were recorded at an average of 18 scans from 4000 to 400 cm^–1^ at a resolution of 4 cm^–1^.

#### 2.8.5 Scanning Electron Microscopy

The microstructure and morphology of nanoliposomes were characterized by using scanning electron microscopy (SEM, Quanta FEG 250 FEI). The freeze-dried samples were mounted onto a metal stub with double-sided carbon conductivity tape and then coated in gold. All SEM pictures were taken with a 5.0 kV accelerating voltage.

### 2.9 Statistical Analysis

All measurements were carried out in triplicate. Experimental data were expressed as mean ± standard deviation (SD). Statistical analysis was carried out using IBM SPSS Statistics 22 (Chicago) software. One-way ANOVA and the Tukey post hoc test were used to compare the treatments, and *p* < 0.05 was taken as a significant value.

## 3 Results and Discussion

### 3.1 Degree of Hydrolysis and Bioactive Characteristics of Hydrolysates

The degree of hydrolysis can be used as an important index to evaluate the extent of enzymatic degradation, as it directly reflects the degree to which protein peptide bonds are broken.^34^ The DH of U. rigida protein hydrolysate (URPH) was found to be 65% after hydrolysis by sequential pepsin and trypsin treatment. This finding is higher than some of the findings from the literature. For instance, Li et al.^35^ stated that the highest DH of Ulva prolifera was approximately 10.60% with alcalase-papain combined treatment. Moreover, Pan et al.^36^ reported that the DH of Enteromorpha clathrata was found to be ∼24.4% by alcalase enzyme. On the other hand, Paiva et al.^24^ obtained the highest DH for U. rigida with bromelain at 86.7%. The differences in the DH values may stem from variations in enzyme specificity and hydrolysis conditions (pH, temperature, time, etc).^37,38^ In the present study, the ACE-inhibitory activity of URPH is determined as 83.10%, whereas that of nonhydrolyzed URPE was 2.90% in our previous study.^23^ Considering the studies on ACE-inhibitory activity of *Ulva* spp. protein hydrolysates, Paiva et al.^24^ and Sun et al.^37^ reported this value as 65.68 and 48.72% for U. rigida and U. intestinalis, respectively. Bioactive peptides embedded in proteins are released by enzymatic hydrolysis and exhibit higher bioactive properties than the protein extract.^39^ The increased activity of the released bioactive peptides has been observed to result in competition with the substrate of the ACE enzyme’s catalytic sites, leading to the inhibition of the enzyme. However, it has been noted that certain types of peptides have the capacity to contribute to a change in the conformation of the enzyme, subsequently leading to a decrease in its activity and resulting in inhibition.^3^

Similarly, in the study by Cermeno et al.,^40^ the ACE-inhibitory activity of protein hydrolysate (36.43%) from Porphyra dioaca was higher than protein extract (14.57%). Moreover, the ACE-inhibitory activity of Actinopyga lecanora was found to be 6.0% before hydrolysis and the highest value at 69.80% after hydrolysis.^41^ The discrepancies among the protein hydrolysates could be attributed to variances in peptide size and composition caused by the specificity of the proteolytic enzymes and other hydrolysis conditions such as time, pH, and temperature.^36,41^

### 3.2 Purification of Bioactive Peptides from Protein Hydrolysate

URPH was subjected to fractionation by ultrafiltration through 3, 5, and 10 kDa membranes. The fractions were coded as UFMS-10 (permeate from 10 kDa), UFMS-5 (permeate from 5 kDa), and UFMS-3 (permeate from 3 kDa) after the ultrafiltration process. Each of these fractions (1 mg/mL) was tested in terms of its ACE-inhibitory activity (Table 1). According to the results, the UFMS-3 fraction had the highest ACE-inhibitory activity, with 77.01%, whereas UFMS-5 showed the lowest activity, with 54.02% (Table 1). Our results were consistent with the findings of the ACE-inhibitory activity of *Ulva* spp. peptides obtained after UFMS.^24,36,37^ According to these reports, peptides with molecular weights below 3 kDa typically exhibit significant ACE-inhibitory activity.

**Table 1:** Angiotensin-Converting Enzyme Inhibitory Activity of the Fractions Obtained after Hydrolysis, Ultrafiltration Membrane Separation, and Ion-Exchange Chromatography^a^

sample	ACE-inhibitory activity (%)
PH	83.10 ± 0.01^a^
UFMS-10	69.01 ± 0.01^e^
UFMS-5	54.02 ± 0.00^f^
UFMS-3	77.01 ± 0.02^c^
IEC-F1	82.03 ± 0.04^b^
IEC-F2	73.02 ± 0.03^d^

a Values are mean ± SD (*n* = 3). Means with different lower-case letters in the column are significantly different (*p* < 0.05). PH: Protein hydrolysate, UFMS-10: the fraction with <10 kDa MW after ultrafiltration membrane separation (UFMS), UFMS-5: the fraction with <5 kDa MW after UFMS, UFMS-3: the fraction with <3 kDa MW after UFMS, IEC-F1: Fraction 1 from ion-exchange chromatography (IEC), IEC-F2: Fraction 2 from IEC.

Then, UFMS-3, showing the highest ACE-inhibitory activity, was subjected to further purification by ion-exchange chromatography (Figure 2). Fraction 1 was collected between 68 and 76 min, while Fraction 2 was collected between 130 and 160 min. Fraction 1 from IEC (IEC-F1, 1 mg/mL) showed the highest ACE-inhibitory activity, as seen in Table 1. Therefore, IEC-F1 was chosen for LC-MS/MS analysis to discover peptides that are potentially related to these bioactivities. The reasons for the increase in ACE-inhibitory activity are discussed in Section 3.3.

**Figure 2:**
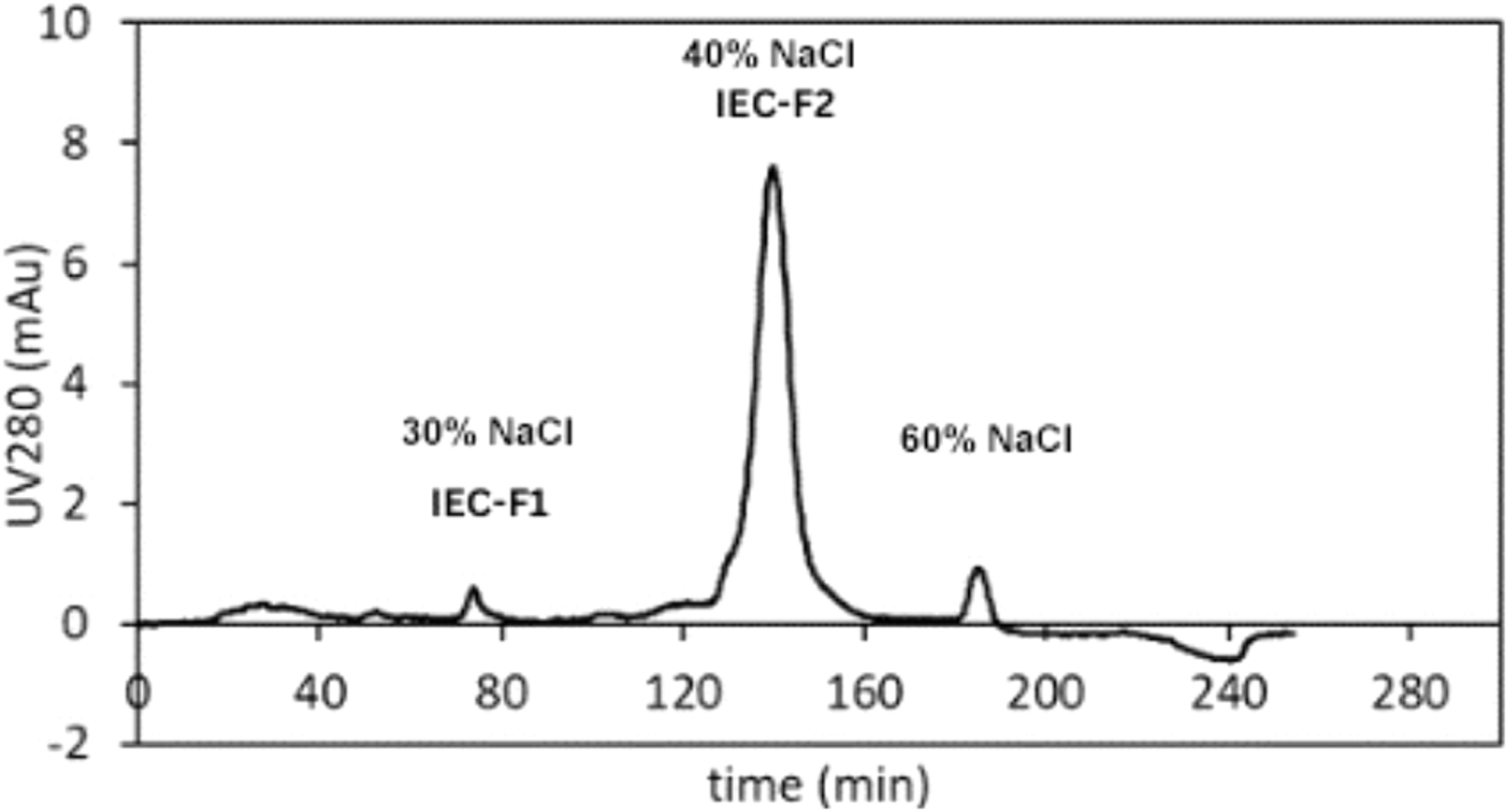
Ion-exchange chromatogram of the fractions. IEC-F1: Fraction 1 from ion-exchange chromatography (IEC), IEC-F2: Fraction 2 from IEC.

### 3.3 LC-MS/MS Analysis of Purified Peptides

Fraction 1, which had the highest ACE-inhibitory activity from the IEC process, was subjected to LC-MS/MS analysis to identify its peptide sequence. According to the results, the sequence of 54 different peptides was elucidated. Among these peptides, in particular, the PeptideRanker score of 21 peptides was >0.50. Then, these sequences were analyzed for their potential bioactivities *in silico* by using the BIOPEP tool.^29^ According to the screening in BIOPEP, 20 ACE-inhibitor peptides were identified (Table 2). WARF, MHPF, and YVGWF are ACE-inhibitor peptides with the highest PeptideRanker scores (>0.95). These peptides had an F residue, being a nonpolar hydrophobic amino acid, at the C-terminal position. Moreover, 9 ACE-inhibitory peptides had the F in the C-terminal (DSDPIEF, GISTMAF, QYASF, GISGTF, MIVF, PIHRSAPAF, IDNIF, and VAGYF) (Table 2). The C-terminal amino acid of the seven ACE-inhibitor peptides in our study (ASANRGIL, RPQL, GVIMIPTL, SDGMPL, QPIADGL, GWKVL, and PKHAPL) was L (Table 2). Similarly, Han et al.^42^ reported that nonpolar hydrophobic amino acids like G, I, L, F, P, W, and Y are essential for the effectiveness of ACE-inhibitors. Especially L, which is a C-terminal residue, may help to block ACE.^37,42^ As seen in Table 2, IDNIF, AQEACWF, and ASANRGIL had I and A residues at the N-terminal. Likewise, Chen et al.^43^ stated that hydrophobic aliphatic branched-chain amino acid residues at the N-terminal position, such as I, L, A, and M were significant in the peptide’s ACE-inhibitor activity. Additionally, RPQL had a basic amino acid (R) at the N-terminal in the present study, and these basic amino acids (R, K, and H) can improve the peptide’s affinity for ACE according to Toopcham et al.^44^ In the literature, several ACE-inhibitory peptides containing 2–7 amino acids were isolated from some green macroalgae like U. intestinal (FGMPLDR and MELVLR), U. rigida (IP and AFL), U. prolifera (WDL, TFDF, WDI, DIGGL, LADAF, and DYLY) by applying different purification methods.^24,35,37^

**Table 2:** Potential ACE-Inhibitory Peptides, as Predicted via PeptideRanker and BIOPEP Tools^a^

peptide sequence	molecular weight (g/mol) (kDa)	PeptideRanker	predicted biological activity
WARF	0.577	0.981525	ACE-inhibitor
MHPF	0.529	0.965807	ACE-inhibitor
YVGWF	0.669	0.96071	ACE-inhibitor
AQEACWF	0.909	0.863384	antioxidant
SDGMPL	0.617	0.832661	ACE-inhibitor
VAGYF	0.554	0.751407	ACE-inhibitor
MIVF	0.507	0.749454	ACE-inhibitor
GISGTF	0.579	0.68639	ACE-inhibitor
GISTMAF	0.724	0.648232	ACE-inhibitor
DSDPIEF	0.820	0.624594	ACE-inhibitor
GWKVL	0.600	0.618744	ACE-inhibitor
NIYVF	0.653	0.609947	ACE-inhibitor, antioxidant
IDNIF	0.619	0.59731	ACE-inhibitor
QYASF	0.613	0.580318	ACE-inhibitor
PIHRSAPAF	0.994	0.573552	ACE-inhibitor
QPIADGL	0.711	0.557233	ACE-inhibitor
PKHAPL	0.660	0.556804	ACE-inhibitor
GYSMAIADF	0.973	0.551983	ACE-inhibitor, antioxidant
ASANRGIL	0.799	0.535836	ACE-inhibitor
GVIMIPTL	0.842	0.531223	ACE-inhibitor
RPQL	0.511	0.50751	ACE-inhibitor

a PeptideRanker: the probability that the peptide exhibits biological activity.

### 3.4 *In Vitro* Cytotoxicity Analysis

The *in vitro* cytotoxicity results are shown in Figure 3. In this test, the cellular viability of normal cells was evaluated after treatment with the IEC-F1 fraction, since this fraction was further utilized for the encapsulation process due to its ACE-inhibitory activity (see Section 3.2). The viability of cells treated with IEC-F1 for 24 h was determined as 58.43, 93.17, and 104.78% for the concentrations of 125, 62.5, and 31.25 μg/mL, respectively. The IEC-F1 concentrations of 62.5 and 31.25 μg/mL showed no cytotoxic effect on L929 cells, while a significant cytotoxic effect was observed at 125 μg/mL (*p* < 0.001) because 70% is considered to be the limiting value in biocompatibility studies.^45^ However, in the study of Li et al.,^35^ peptides from U. prolifera tested on HUVEC (human umbilical vein endothelial) cells had a noncytotoxic effect for 12 h. Ebrahimi et al.^46^ reported that Spirulina platensis-derived peptides had no toxic effect on HFFF-2 (human foreskin fibroblast) cells within 48 h. In addition, Kose and Oncel^47^ reported that the reduction in cell viability was lower than 50% at high S. platensis peptide concentrations (800 μg/mL) while cell viability increased between peptide concentrations of 200 to 800 μg/mL.

**Figure 3:**
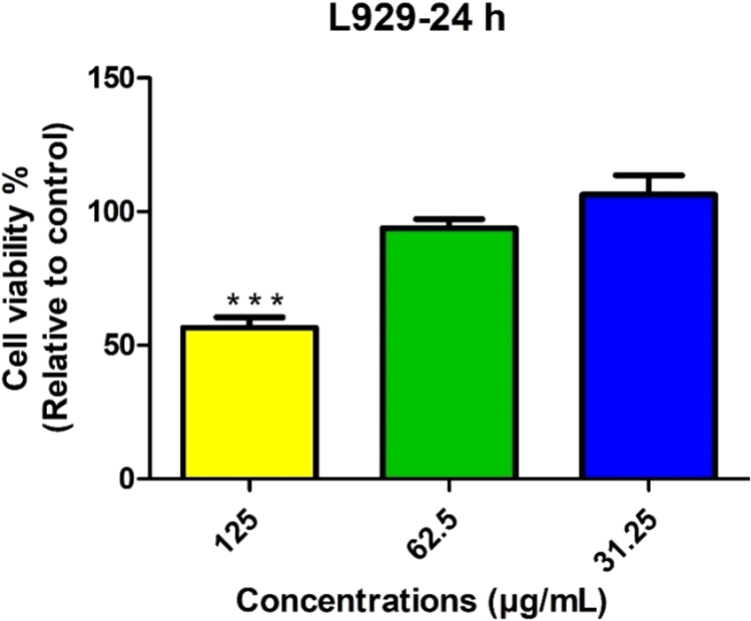
Effect of the fraction (IEC-F1), obtained after ion-exchange chromatography, on cellular viability (%) of a healthy mouse fibroblast cell line (L929). *p* < 0.001***: shows the statistical significance values between groups.

### 3.5 Nanoencapsulation of Bioactive Peptides

#### 3.5.1 Physical Properties of Nanoliposomes

Peptide fractions (IEC-F1) were encapsulated in a nanoliposome system based on a thin-film hydration technique and coated with chitosan to increase the stability. The ζ-potential, mean particle size, and PDI of chitosan-coated and noncoated nanoliposomes loaded with IEC-F1 are shown in Table 3. These factors were evaluated as indicators of the physical stability of the nanoliposomes.

**Table 3:** Physical Properties and Encapsulation Efficiency of Chitosan-Coated and Noncoated Nanoliposomes Containing IEC-F1^a^

chitosan (w/v %)	mean particle size (nm)	polydispersity index	ζ-potential (mV)	encapsulation efficiency (%)
control*	73.79 ± 0.02^b^	0.28 ± 0.01^b^	–6.16 ± 0.02^b^	
0	76.95 ± 0.68^b^	0.26 ± 0.02^b^	–4.53 ± 0.69^b^	93.03 ± 0.80^a^
0.6	334.84 ± 7.80^a^	1.00 ± 0.01^a^	26.70 ± 1.79^a^	92.01 ± 4.51^a^

a Values are mean ± SD (*n* = 3). Superscript letters (a, b) are significantly different (*p* < 0.05). *Control: empty nanoliposomes.

The ζ-potential is a measure of the surface electric charge of particles, an important parameter that affects liposome stability.^48^ It is generally assumed that a surface charge of ±30 mV is required for stable dispersion.^49^ The ζ-potentials of empty nanoliposomes and nanoliposomes loaded with IEC-F1 (0.14 mg/mL) were found to be −6.16 ± 0.02 and −4.53 ± 0.69 mV (*p* > 0.05, Table 3), respectively. Similarly, da Rosa Zavareze et al.^48^ reported that ζ-potential values of nanoliposomal croaker protein hydrolysates were found to be −5.5 mV. However, in the present study, lower negative ζ-potential values for the liposomes after being loaded with IEC-F1 (−4.53 ± 0.69 mV) were determined compared to those of food peptide-loaded nanoliposomes, which range between −8.3 and −40.8 mV according to Taylor et al.^50^ and Mosquera et al.^51^ This difference may stem from NaCl molecules in IEC-F1 after ion-exchange chromatography. Likewise, it has been reported that the presence of NaCl solution in the medium causes a decrease in the ζ-potential of nanoliposomes.^32^ This wide variety of ζ-potentials can be linked to variations in solution conditions (such as pH and ionic strength), as well as variations in the type, content, and purity of the phospholipid material.^48,49^ In addition, the ζ-potential values of protein hydrolysates differ depending on the enzymes used in the hydrolysis process due to differences in the compositions and release of some charged amino acids.^52^ After chitosan coating, anionic nanoliposomes exhibited cationic properties (26.7 ± 1.79 mV). Similarly, Ma et al.^32^ reported that the ζ-potential of the nanoliposomes varied between 22.03 mV and 28.03 mV after chitosan coating. Sarabandi and Jafari^10^ stated that the ζ-potential of the nanoliposomes ranges from 6.73 to 32.52 mV after chitosan coating. Chitosan coating causes the ζ-potential of the nanoliposomes to change from negative to positive charge, which is due to the binding of positively charged chitosan molecules to the negatively charged particles.^53^

The mean particle size of the encapsulate has a significant effect on the release and digestibility of the bioactive compound. In food applications, smaller particle diameter improves solubility, bioavailability, and controlled release of the bioactive compound due to increased surface area.^54^ The mean particle size of chitosan-coated and noncoated nanoliposomes loaded with IEC-F1 was found to be 73.79 ± 0.02 and 76.95 ± 0.68 nm (Table 3). After loading, there was no statistically significant increase in particle size (*p* > 0.05). The small increase in particle size can be explained by the low molecular weight of the peptide used, as seen in Table 2. Hosseini et al.^55^ reported that the molecular weight and concentration of peptide fractions loaded into nanoliposomes had an impact on nanoliposome size. Nanoliposomes coated with 0.6% chitosan were characterized by a particle size of 334.84 ± 7.80 nm (Table 3). Recent investigations have shown that chitosan coating generally causes an increase in particle size.^10,32,56^ The lipid–chitosan electrostatic interactions between the phospholipid head groups and the particular functional groups (NH_3_^+^) of chitosan can be used to explain the increase in particle size.^57^

There was no statistical difference between the empty and IEC-F1-loaded nanoliposomes, and PDI values were 0.28 and 0.264, respectively (*p* > 0.05). This value is consistent with the findings of Sarabandi and Jafari^10^ on chitosan-coated nanoliposomes loaded with flaxseed-peptide fractions; the PDI of empty and peptide-loaded nanoliposomes was reported to be 0.284 and 0.326, respectively. PDIs between 0.33 and 0.44 demonstrated that the particle size is confined within a small range.^9,56^ A low PDI value indicates homogeneity in the particle size distribution.^48^ Large PDI values are most likely the result of particle aggregation and precipitation inside the system.^9,48^ In this study, PDI values increased after being coated with chitosan (Table 3). Similarly, Li et al.^9^ reported that the PDI of nanoliposomes coated with chitosan was higher than uncoated nanoliposomes. Different materials decorated on liposomes can have different effects on size, polydispersity, and ζ-potential.^58^

#### 3.5.2 Encapsulation Efficiency

The encapsulation efficiency of chitosan-coated and noncoated nanoliposomes loaded with IEC-F1 was found to be 92.01 and 93.03%, respectively (*p* > 0.05, Table 3). In comparison to other studies, the EE values obtained in this study were higher than those of flaxseed-derived bioactive peptides (between 84 and 87%),^10^ rainbow trout skin peptides (between 68.5–80.2),^56^ salmon hydrolysate (73%),^9^ and white croaker fish hydrolysates (80%).^48^

The ζ-potential of chitosan-coated and noncoated nanoliposomes loaded with IEC-F1 were −4.53 and 26.70 mV, respectively. For a high encapsulation efficiency, the electrostatic repulsion between the particles will typically be strong enough to prevent aggregation when the particles have a greater absolute value of ζ-potential of >30 mV.^9^ On the other hand, chitosan coating was insignificant to the encapsulation efficiency of the nanoliposomes (*p* > 0.05, Table 3). The fact that the encapsulation efficiency was not increased by the addition of chitosan may be related to the cationic charges in chitosan competing with the cationic charges in the peptides in the liposome, and therefore, more peptides may have replaced chitosan.^59^

#### 3.5.3 Characterization of Nanoliposomes

##### 3.5.3.1 Thermal Properties

Thermal properties of the nanoliposomes were determined by DSC to assess the influence of heating on the chitosan-coated liposomal system and gain further insight into the interactions of the charged peptide fraction with the lecithin bilayers. The denaturation temperatures of chitosan and chitosan-coated empty liposomes were 166.5 ^60^ and 127.44 °C. In the present study, denaturation temperature and enthalpy of chitosan-coated nanoliposomes loaded with IEC-F1 were found to be 77.41 °C and 197.91 J/g, respectively (Figure 4). In contrast, in the study of encapsulation of fish-derived peptides with chitosan-coated liposomes by Ramezanzade et al.,^61^ the denaturation temperature of peptides was found to be 168.7, 237.3, and 306.5 °C. Denaturation temperature and denaturation enthalpy can vary depending on the structural properties of the protein and experimental conditions. For instance, the pH at which encapsulation takes place and low pH conditions, where protein solubility decreases, may also have affected the thermal stability of the peptide.^62^ Sreedhara et al.^63^ examined the thermal stability of lactoferrin at various pH values and found that the stability decreased with decreasing pH. Additional factors such as hydrophobic packaging, polar/nonpolar ratios, disulfide bond strength, charge–charge balances, interactions with helical dipoles, entropic stabilization, molecular flexibility, and amino acid content are some structural properties that may affect the stability of folded proteins.^64^

**Figure 4:**
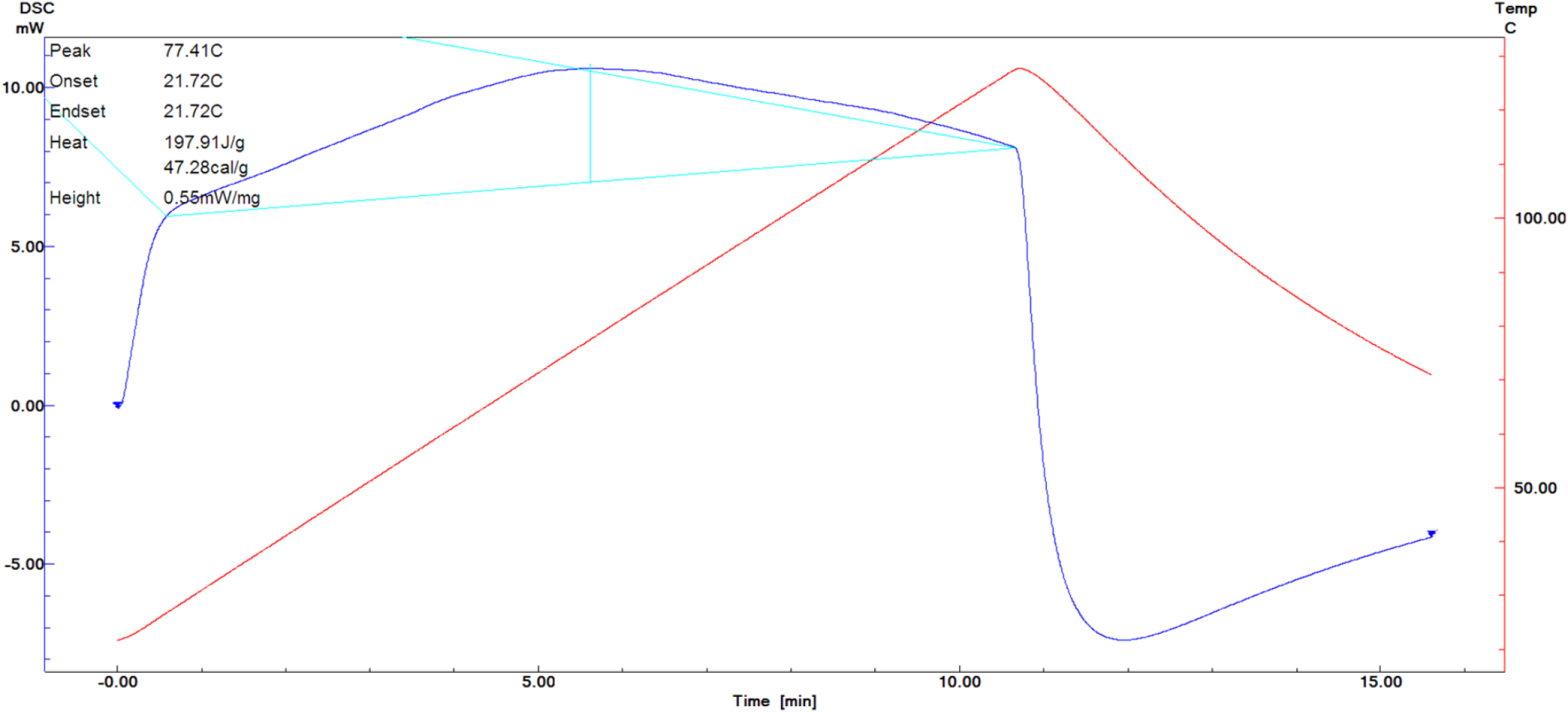
DSC of chitosan-coated nanoliposomes loaded with IEC-F1.

##### 3.5.3.2 FT-IR

The FT-IR spectra of the chitosan-coated nanoliposomes loaded with IEC-F1 are listed in Figure 5. In general, the basic ranges in the chemical structure of proteins are associated with the O–H stretch (3737 and 2933 cm^–1^), N–H stretch (3431 cm^–1^), C=O stretch, amide-I region (1647 cm^–1^), C–N stretch and N–H deformation (1537 cm^–1^), C=O stress (1071 cm^–1^), and N–H bending (670 cm^–1^).^52,65^

**Figure 5:**
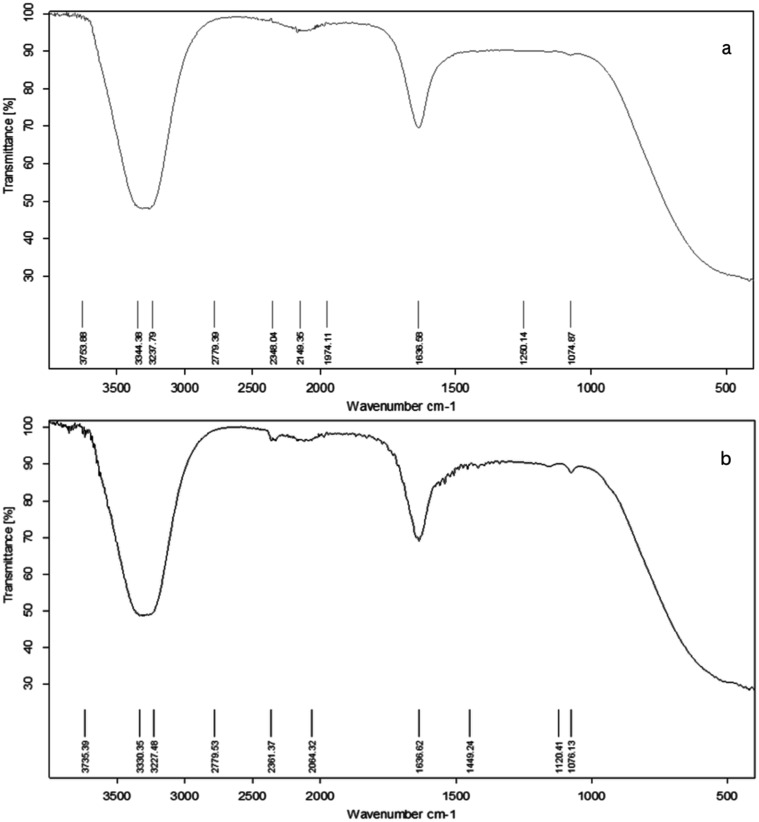
(a) FT-IR spectrum of chitosan-coated nanoliposomes (control) and (b) chitosan-coated nanoliposomes loaded with IEC-F1.

In the present study, the location of the hydrogen bond between the chitosan’s hydroxyl/amino groups and the peptide’s carboxylic acid/amino groups shifted from 3254 to 3330 cm^–1^ after the encapsulation.^66^ The peak detected at ∼1636 cm^–1^ represents the C=O stretch of the ester bond where the hydrocarbon chain meets the headgroup in the lecithin molecule (Figure 5a,b). The band was found at 1440 cm^–1^, which corresponds to the stretching vibrations of alkene CH_2_ (Figure 5).^56^ This likely demonstrates the complex interactions of lecithin molecules embedded in liposomes with C–S molecules.^56^ The band was visible at 1074 cm^–1^ attributed to the symmetrical stretching of PO_2_ (Figure 5b). This band is due to the formation of electrostatic interactions between phosphoric groups of lecithin and amine groups of chitosan.^67^ The intermolecular interactions between the amino groups of the peptide fraction and the functional groups of chitosan caused these modifications. This indicates that the loading of peptide fractions into liposomes is connected to the localization inside lipid bilayers.^68^

##### 3.5.3.3 Morphological Properties

Morphological structures are essential indicators of physicochemical and functional properties, stability, particle size, and distribution of nanoliposomes.^52^ The structural properties of chitosan-coated nanoliposomes loaded with IEC-F1 are shown in Figure 6. The nanoliposomes were found to have smooth and spherical surfaces in the SEM images (Figure 6b).

**Figure 6:**
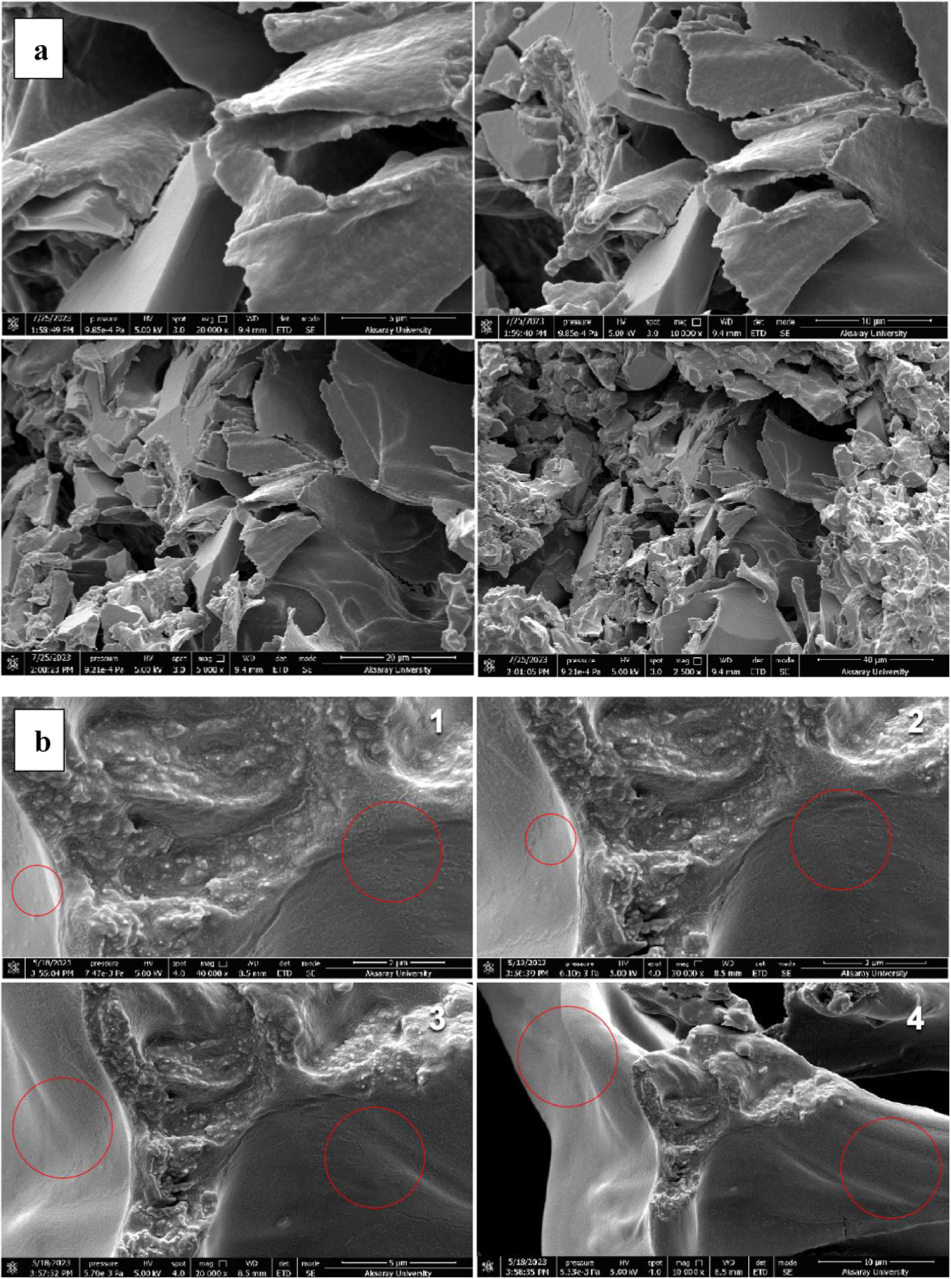
(a) Morphological properties of freeze-dried chitosan-coated nanoliposomes (control) and (b) morphological properties of freeze-dried chitosan-coated nanoliposomes loaded with IEC-F1.

Spherical particles offer a stronger controlled release ability and preserve the encapsulated substances because they have less surface contact with the environment. Similarly, Li et al.^9^ reported that the morphological structure of chitosan-coated nanoliposomes loaded with peptides derived from salmon protein consisted of spherical particles. When the particle size and PDI values of the chitosan-coated nanoliposomes loaded with IEC-F1 were examined, it was seen that the PDI value and particle size were especially high (Table 3). This showed that there was too much aggregation in the media. The SEM image of the chitosan-coated nanoliposomes loaded with IEC-F1 also supported this finding (Figure 6b). According to Ma et al.,^32^ the outer film thickness and number of layers of liposomes increased after coating with chitosan. Additionally, it has been demonstrated that chitosan is absorbed by the outer surface of the liposomes, increasing their thickness and particle size. They hypothesized that the chitosan-coated liposomes cluster with one another, leading to the noticeably larger average size.^32^ In conclusion, the effect of preparation method, formulation type, and composition on the physical, functional, performance, and stability qualities of the generated capsules is reflected in the morphological structure of the carriers.^52^

##### 3.5.3.4 ACE-Inhibitory Activity of Nanoliposomes

In the present study, the ACE-inhibitory activity of unencapsulated IEC-F1 was found to be 82.03% (Table 1). The IEC-F1 showed 29.8% of ACE-inhibitory activity after encapsulation. Similarly, Correa et al.^69^ stated that the ACE-inhibitory activity of unencapsulated peptides (79%) showed higher activity than that of encapsulated peptides (54%). Marin-Penalver et al.^70^ showed that the ACE-inhibitory activity of collagen hydrolysate liposomes was slightly increased compared to collagen hydrolysate. During the encapsulation process, sonication, which was applied to decrease in size of the particles, may have affected the bioactivity of the encapsulated peptides.^69^

## 4 Conclusions

In this study, U. rigida proteins were subjected to enzymatic hydrolysis and then separated into fractions using ultrafiltration membranes according to their molecular weights. Molecular weights affected the ACE-inhibitory activity, and it was determined that especially fractions with a molecular weight of <3 kDa showed the highest ACE-inhibitory activity. These <3 kDa fractions were further purified according to their ionic charges by ion-exchange chromatography. Among the fractions obtained as a result of purification, the highest ACE-inhibitory activity was measured as 82% (IEC-F1). A total of 21 ACE-inhibitory peptides were identified, with a maximum ACE inhibition of 82.03%. *In vitro* cytotoxicity analyses indicated that the peptide solution (IEC-F1) had no toxic effects. Moreover, bioactive peptides from U. rigida (IEC-F1) were encapsulated by liposomal encapsulation. These findings highlight that the applied process has promising potential for the production and purification of ACE-inhibitory peptides from U. rigida. Therefore, regardless of the need for further investigation, U. rigida demonstrates a high potential as a source of ACE-inhibitory peptides in functional food and drug formulations. Encapsulation with chitosan-coated nanoliposomes offers a potential carrier system for functional food and nutraceutical applications of these peptides. In future studies, ACE-inhibitory activity can be precisely established and verified using synthetic peptides with identical sequences and evaluated with *in vivo* studies.
